# Supplementary material for: Patterns of Intron Gain and Loss in Fungi
Source: PLoS Biol. 2004 Nov 30;2(12):e422. doi: 10.1371/journal.pbio.0020422 (PMC532390; doi:10.1371/journal.pbio.0020422)
Supplement: Table S1 — Also available at http://genes.mit.edu/NielsenEtAl/. (4.3 MB ZIP). [file pbio.0020422.st001.zip › NielsenEtAl/html/900.html]

AN0447.1.NCU01779.1.MG06993.1.FG00654.1


```
 CLUSTAL W (1.82) Multiple Sequence Alignments - Introns Inserted


Sequence 1: MG06993.1	323 aa
Sequence 2: FG00654.1	304 aa
Sequence 3: NCU01779.1	326 aa
Sequence 4: AN0447.1	326 aa
Alignment Length: 339 aa
Number Identitical Residues: 153 aa
Alignment Score (without introns) 6657


MG06993.1 	MTATKLLRTS--MSVLWR---PRQVRTS-AVTYSICNLATRR-AVHSSSK-----PLWAV
NCU01779.1	MPLNRLLLRKGLVPLVSK---STVRPAATTIPCLRQGAAASAPASTSTSSRQAHTGARSA
FG00654.1 	MASSRTMSRT--LAALTR---PVTETSSRAVPRWTRSMATVRSRIPSISS-----GLRPL
AN0447.1  	MASSSTSANMVKRTILSNRLAAQILNFAEASPRTIRPFAFGSIRSIHNSARLPTITASET
          	*. .         . : .  :.     : : .      *         *   .       

MG06993.1 	VPR---RPKLQTQQLRGGISGGADSRRTIFIQTEPTPNSD~ALKFLPNHRVLPEDISTPF
NCU01779.1	ARP---RLQQRSWGALNTTLAPQQQRRTIFIQTENTPNPD~SLKFLPNQKVIPDSIKTPF
FG00654.1 	AQR---QRIVRTATPS--LAGIGSGVRTIFIQTENTPNPD~ALKFLPNHRVVPEEFSTPF
AN0447.1  	RHRPTLRPHQLSAAHGRVNGQPPTGKRTIFIQTESTPNPD0ALKFIPNHRVLPEDFPTSF
          	   .: :    :              ******** ***.* :***:**::*:*:.: *.*

MG06993.1 	IEYLNPRSTIAPPYPSPLAAQLMNIDGVQSVFYGADFITVTKASDANWAHIRPEIFSLIT
NCU01779.1	IEYMNARSTIAPPYPSPLAAQLMNIEGVTSVFYGTDFITVTKSADANWAHIRPEVFALIT
FG00654.1 	IEYLNPRATISPPHPSPLAAKLMNIDGVTSVFYGADFITVTKAADANWAHIRPEIFALIT
AN0447.1  	LEYLSPRSTLAPPHPSTLAANLFNVEGVQSVFFGTDFITVTKASDTNWAHIKPEVFSLIT
          	:**:..*:*::**:**.***:*:*::** ***:*:*******::*:*****:**:*:***

MG06993.1 	EAITSGQKIVNIVERTDASGEDGQETSGEVDSLSYNENDSEVVGMIKELLETRIRPAIQE
NCU01779.1	ETITSGQTIVNVVERNE--GEESTQESDEKDSLAYDENDSEVVGMIKELLETRIRPAIQE
FG00654.1 	EAITAGEQIVTISERRE--GEAG--APVEEDSLAYNENDSEVVGMIKELLETRIRPAIQE
AN0447.1  	QAVTSGEPIVNTVEKSGASGQKG----GEEDSLSYNEEDDEVVSMIKELLETRIRPAIQE
          	:::*:*: **.  *:  ::*: .     * ***:*:*:*.***.****************

MG06993.1 	DGGDIEFRGFED-GNVLLKLRGACRTCDSSTVTLKNGIEGMLMHY0HFGQIEEVQGVIQV
NCU01779.1	DGGDIEFRGFED-GIVKLKLRGACRTCDSSTVTLKNGIEGMLMHY0----IEEVQGVEQV
FG00654.1 	DGGDIDFRGFDDEGYVHLRLRGACRTCDSSTVTLKNGIEGMLMHY0----IEEVKGVKQV
AN0447.1  	DGGDIEFRGFEN-GIVMLKLRGACRTCDSSTVTLRNGIESMLMHY0----IEEVQGVEQV
          	*****:****:: * * *:***************:****.*****     ****:** **

MG06993.1 	MDPEEEIAAKEFAKFEEKLRAQKGEAPPSTVGKGSLDTVSG
NCU01779.1	LDPEEDIALQEFQKFEEKLRKQKGEVPPTTVGKDSLDSVPG
FG00654.1 	MDQEEEIALQEFEKFEEKLKQQKGQAASA------------
AN0447.1  	LDEEEEISMLEFAKFEEKLRQQKGAQATAPS---SLDSAP-
          	:* **:*:  ** ******: ***  ..:.    : .: .
```
